# Cognitive, Mental Health, Functional, and Quality of Life Outcomes 1 Year After Spontaneous Subarachnoid Hemorrhage: A Prospective Observational Study

**DOI:** 10.1007/s12028-023-01895-y

**Published:** 2023-12-21

**Authors:** Verena Rass, Klaus Altmann, Laura Zamarian, Anna Lindner, Mario Kofler, Max Gaasch, Bogdan-Andrei Ianosi, Lauma Putnina, Philipp Kindl, Margarete Delazer, Alois J. Schiefecker, Ronny Beer, Bettina Pfausler, Raimund Helbok

**Affiliations:** 1 Department of Neurology, Medical University of Innsbruck, Innsbruck, Austria; 2 Department of Anaesthesiology and Intensive Care Medicine, Medical University of Innsbruck, Innsbruck, Austria; 3 Department of Neurology, Johannes Kepler University Linz, Krankenhausstraße 7a, 4020 Linz, Austria

**Keywords:** Subarachnoid hemorrhage, Neuropsychological evaluation, Cognitive deficits, Mental health outcomes, Quality of life

## Abstract

**Background:**

Patients with spontaneous subarachnoid hemorrhage (SAH) frequently encounter cognitive dysfunction and mental health issues with negative effects on health-related quality of life (HR-QoL). Here, we aimed to describe the prevalence of cognitive deficits, mental health problems, and HR-QoL impairments 1 year after SAH.

**Methods:**

In this prospective observational study, 177 patients with SAH admitted to our neurointensive care unit over a time span of ten years followed the invitation for an in-person 1-year follow-up, including a standardized neuropsychological test battery. Mental health issues (anxiety and depression) and HR-QoL were evaluated using questionnaires (Hospital Anxiety and Depression Scale; 36-item Short Form questionnaire). Functional outcome was assessed with the modified Rankin Scale (mRS) score.

**Results:**

Patients were 54 years of age (interquartile range 47–62 years) and presented with a median Hunt and Hess score of 2 (interquartile range 1–3) at admission. Most patients (93%) achieved good functional 1-year outcomes (mRS score 0–2). Seventy-one percent of patients had deficits in at least one cognitive domain, with memory deficits being the most prevalent (51%), followed by deficits in executive functions (36%), visuoconstruction (34%), and attention (21%). Even patients with perimesencephalic SAH (18%) or with full functional recovery (mRS score = 0, 46%) had a comparable prevalence of cognitive deficits (61% and 60%, respectively). Symptoms of depression and anxiety were reported by 16% and 33% of patients, respectively. HR-QoL was impaired in 37% (55 of 147). Patients with cognitive deficits (*p* = 0.001) or mental health issues (*p* < 0.001) more frequently reported impaired HR-QoL.

**Conclusions:**

Most patients with SAH have cognitive deficits and mental health issues 1 year after SAH. These deficits impair patients’ quality of life.

**Supplementary Information:**

The online version contains supplementary material available at 10.1007/s12028-023-01895-y.

## Introduction

Although mortality has declined substantially after spontaneous subarachnoid hemorrhage (SAH) in the past decades, case fatality rates remain high, ranging between 18 and 43% after the year 2000 [1]. With an average age at disease onset between 50 and 60 years, many patients are at the peak of their professional career and play an indispensable role for their family and socioeconomic environment. Using conventional outcome parameters, the majority of survivors (up to 60%) achieve favorable outcomes after SAH [2]. However, cognitive impairments are evident even in patients with good functional appearance and may remain unrecognized when only assessing functional outcome scores such as the modified Rankin Scale (mRS) score or the Glasgow Outcome Score. Numbers of patients with SAH who subsequently exhibit cognitive impairments are reported to be as high as 40–70% [3, 4]. Persisting cognitive deficits can have effects on day-to-day and social life and negatively influence patients’ activities of daily living, return-to-work, and health-related quality of life (HR-QoL) [5, 6]. To provide targeted cognitive rehabilitation strategies and facilitate workplace reintegration, identification of domain-specific cognitive deficits is crucial. Similarly, mental health and psychosocial problems, including depression and anxiety, affect almost every second patient 1 year after aneurysmal SAH based on pooled frequencies [4]. These symptoms substantially affect patients’ quality of life (QoL) [5].

In this prospective observational study, we aimed to describe multidimensional outcomes, including domain-specific cognitive functions, mental health, and HR-QoL, 1 year after spontaneous SAH. Furthermore, we aimed to identify factors that are associated with cognitive deficits. We hypothesized that cognitive deficits are prevalent and influence functional outcome and HR-QoL 1 year after SAH.

## Methods

### Study Design, Setting, and Participants

For this observational cohort study, patients with spontaneous SAH admitted to the neurological intensive care unit (ICU) of a tertiary hospital (Medical University of Innsbruck) were screened between April 2010 and December 2020. Of 425 patients, 177 patients fulfilled the following inclusion criteria: (1) diagnosis of a spontaneous aneurysmal and non-aneurysmal SAH (grades 1 to 5 on the Hunt and Hess [H&H] scale) confirmed by computed tomography (CT) scan or lumbar puncture, (2) age greater or equal to 18 years, (3) ICU stay for more than 24 h, (4) fluent in the German language, and (5) neuropsychological and clinical examination at the 1-year follow-up. Supplemental Fig. 1 shows the patient selection by a flowchart. The local institutional review board (Medical University of Innsbruck, AM4091-292/4.6) granted approval for this study. All patients provided informed consent according to local regulations in accordance with the Declaration of Helsinki. Results are reported based on the Strengthening the Reporting of Observational Studies in Epidemiology statement.

**Fig. 1:**
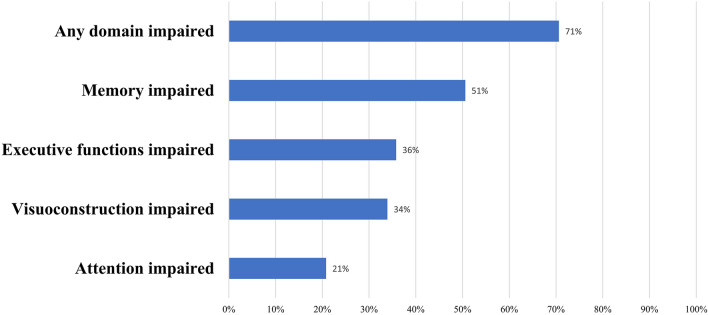
Domain-specific cognitive deficits (percentage) 1 year after subarachnoid hemorrhage in 177 patients

### Clinical Management

All patients received treatment according to evidence-based international guidelines (except for nimodipine being administered intravenously in poor-grade patients) [7–9]. Prophylactic nimodipine treatment was administered in all patients. Treatment decisions regarding endovascular coiling or neurosurgical clipping for early aneurysm occlusion were based on interdisciplinary case-by-case discussions. Repetitive transcranial color-coded duplex sonography (LOGIQ S8; GE Healthcare, Chicago, IL) was performed to detect large-vessel sonographic vasospasm, which was defined as an elevation of mean velocities > 120 cm/s in the middle or anterior cerebral artery or a daily change in mean velocities > 50 cm/s. In the setting of severe vasospasm, intra-arterial nimodipine was administered after confirmation by catheter cerebral angiogram. Delayed cerebral ischemia was defined as neurological deterioration (new focal neurological deficits and/or a ≥ 2-point decline on the Glasgow Coma Scale) and/or new ischemic lesions on follow-up imaging (CT or magnetic resonance imaging) not attributable to any other cause [10].

Neurorehabilitation after acute ICU treatment followed a multimodal concept. The stages of treatment extend from phase A, the acute phase, to phase E, in which social and professional reintegration takes place. The goal setting was adapted to the individual needs of the patients. According to local regulations, patients received a maximum of 3 h of therapy on working days, and the overall rehabilitation time conformed to the patient’s needs [11].

### Outcome Instruments

Functional outcome was assessed at discharge and 3 and 12 months after bleeding using the mRS. Favorable outcome was defined as mRS scores 0–2. At the 1-year follow-up, each patient underwent a medical examination by a doctor of the study team.

All included patients underwent detailed neuropsychological testing performed by experienced neuropsychologists who were blinded to the clinical course. Using a standardized test battery, several cognitive domains were screened as follows:

1. Executive function was evaluated using six different tests: the Frontal Assessment Battery, which consists of six subtests exploring conceptualization, phonematic verbal fluency, motor programming, sensitivity to interference, inhibitory control, and environmental autonomy [12, 13]; the semantic verbal fluency test (animals per minute), or the Regensburger Verbal Fluency Test (Regensburger Wortflüssigkeitstest; RWT), to assess executive language functions [14]; the Trail Making Test (TMT) A and B to examine processing speed and mental flexibility [15]; the digit span backward subtest of the Wechsler Memory Scale-Revised to assess verbal working memory [16]; and a clock drawing task (CLOX) to assess figure planning [17]. The TMT-B was adjusted for education years with normative values. Impairment of executive function was assumed if the patient scored below the tenth percentile (according to age and/or education stratified norms) in two of six tests.
2. To assess memory function, two important measures were used: (1) the delayed recall subtest of the Verbal Learning and Memory Test (Verbaler Lern- und Merkfähigkeitstest; VLMT) [18] and (2) the delayed recall subtest of the Rey–Osterrieth complex figure test (ROCF) [19]. Impairment in memory function was assumed if the patient scored below the tenth percentile (according to age stratified norms) in one of two tests.
3. Attention deficits were assumed if the patient scored below the tenth percentile (according to age norms) in the digit span forward subtest of the Wechsler Memory Scale-Revised [13].
4. Deficits in visuoconstruction were assessed with the copy subtest of the ROCF [19] and scored in the setting of scores below the tenth percentile.
5. Overall cognitive functions were screened using the Mini-Mental State Examination, and impairment was classified in patients who scored ≤ 24 out of 30 points [20].

The Hospital Anxiety and Depression Scale (HADS) was applied to detect symptoms of anxiety and depression in survivors of SAH [21]. It consists of an anxiety (HADS‐A) and depression (HADS‐D) subscale, each testing seven items scored from 0 to 3. Scores range from 0 to 21 in each subscale. Lower scores are linked to less severe anxiety‐ and depression‐related symptoms. Scores > 7 suggest anxiety disorder or depression.

HR-QoL was assessed with the 36-item Short Form (SF-36). The SF-36 is a self-report questionnaire and rates the subjective health condition [22]. It provides scores of eight health domains, which can be classified into the physical component summary and mental component summary, each ranging from 0 to 100 points. Higher levels indicate a better health condition. Scores below 40 are considered impaired according to norm-based scoring.

### Statistical Analysis

All statistical analyses were generated using IBM SPSS Statistics for Windows (IBM Corp., released 2020, Version 27.0; IBM Corp, Armonk, NY). Numerical data were assessed for normality and are given as medians and interquartile ranges (IQRs); categorical data are reported as counts and proportions. Univariate analysis was done with use of the *t*-test or Mann–Whitney *U*-test for continuous variables and Fisher’s exact test for categorical variables, as appropriate. To identify clinically relevant risk factors assessed during the acute phase of disease for cognitive impairments, significantly associated factors (*p* < 0.1) in univariate analysis were included stepwise in multivariable logistic regression models using generalized linear models and were retained if significant (*p* < 0.05). Independent associations between cognitive/mental health status and functional outcome at the 1-year follow-up were calculated with generalized linear models adjusted for the H&H score on admission and age as established outcome parameters. The mRS score at 12 months served as an ordinal dependent variable. To check for changes in the mRS score between discharge and the 1-year follow-up, the McNemar test was used.

## Results

### Patient Population

Of 425 patients, 177 patients were able to complete the 1-year neuropsychological evaluation in-person. At ictus, patients had a median age of 54 (IQR 47–62) years, and 105 (59%) were women (Table 1). The median H&H grade at admission of 2 (IQR 1–3) was significantly lower compared with that for the excluded patient group, who died or did not undergo neuropsychological testing (H&H grade 3 [IQR 1–5], *p* < 0.001), indicating a less severely injured subpopulation. Still, patients of all disease severity groups were included in the final analysis (Table 1). Included patients were younger (*p* < 0.001; Supplemental Table 1). The diagnosis of dementia was not recorded in any patient. The median follow-up interval from ICU admission was 374 (IQR 365–387) days.

**Table 1:** Demographics of 177 patients with subarachnoid hemorrhage

	*N* = 177
Baseline characteristics	
Age, years	54 (47–62)
Female sex	105 (59.3%)
Hypertension history	69 (38.9%)
Diabetes mellitus II	8 (4.5%)
Smoking history	72 (40.7%)
Years of education	10 (9–12)
Admission variables	
Loss of consciousness at ictus	51 (28.8%)
Parenchymal bleeding at admission	28 (16.1%)
Hunt and Hess Score at admission	2 (1–3)
1	59 (33.3%)
2	52 (29.4%)
3	32 (18.1%)
4	12 (6.8%)
5	22 (12.4%)
Modified Fisher Scale at admission	3 (2–4)
SEBES score at admission	1 (0–2)
Hjidra score	11 (6–18)
Hjidra ventricle score	1 (0–4)
Aneurysm location	
Anterior circulation	83 (46.9%)
Posterior circulation	42 (23.7%)
No aneurysm	51 (28.8%)
Unknown/other	1 (0.6%)
Aneurysm treatment	
Coiling	88 (49.7%)
Clipping	39 (22.0%)
No intervention	51 (28.8%)
Hospital complications	
Hydrocephalus requiring EVD	71 (40.1%)
Large-vessel vasospasm	81 (45.8%)
Delayed cerebral ischemia	22 (12.4%)
Ventriculitis	21 (11.9%)
Pneumonia	61 (34.5%)
Urinary tract infection	41 (23.2%)
Sepsis/bacteremia	18 (10.2%)
Outcomes	
Length of ICU stay, days	17 (10–27)
Hospital mortality	0 (0%)
mRS at discharge	2 (1–4)
mRS at 3 months	1 (0–2)
mRS at 12 months	1 (0–2)
0	82 (46.3%)
1	47 (26.6%)
2	35 (19.8%)
3	9 (5.1%)
4	3 (1.7%)
5	1 (0.6%)

Data are *n* (%) or median (interquartile range)

EVD, external ventricular drain, ICU, intensive care unit, mRS, modified Rankin Scale score, SEBES, subarachnoid hemorrhage early brain edema score

### Cognitive Outcomes 1 Year After SAH

**Fig. 2:**
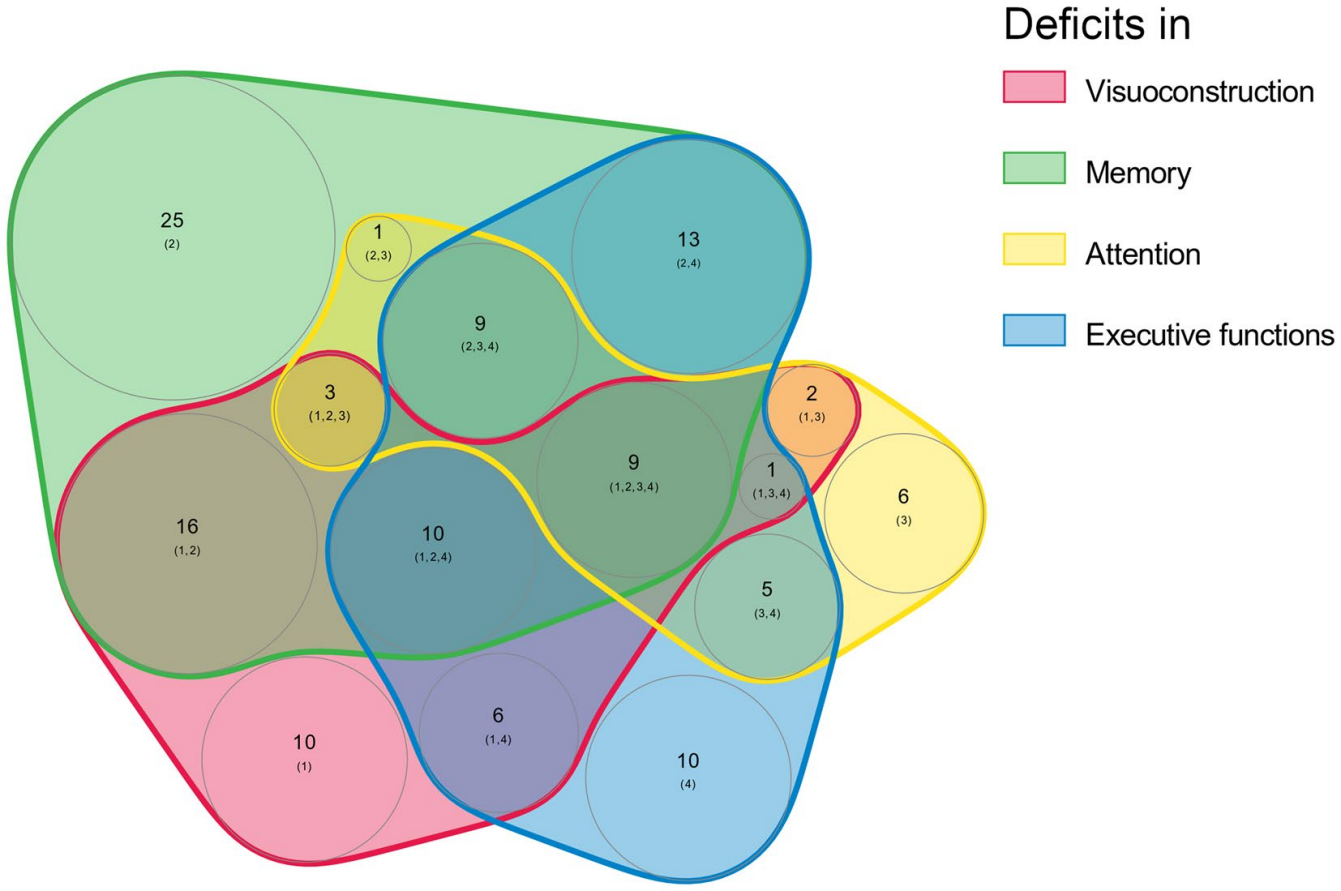
Number of patients with co-occurrence of multiple cognitive deficits versus single occurrence of one domain-specific cognitive deficit

Test results of single examinations 1 year after SAH are given in Table 2. Overall, 71% (125 of 177) of patients had deficits in at least one cognitive domain, with memory deficits being the most prevalent (51%), followed by deficits in executive functions (36%), visuoconstruction (34%), and attention (21%; Fig. 1). More precisely, verbal memory was impaired in 37% (VLMT delayed recall), and deficits in visual memory were evident in 30% of patients (ROCF delayed recall). Executive dysfunctions comprised deficits in cognitive flexibility (27%, TMT-B), deficits in working memory (23%, WMS backward), decreased processing speed (21%, TMT-A), and decreased semantic verbal fluency (13%, RWT, animals/minute). Although co-occurrence of multiple cognitive deficits was frequent (7% of patients with deficits in all tested domains), other patients had deficits in a single domain only (Fig. 2).

**Table 2:** Neuropsychological test performance 1 year after subarachnoid hemorrhage in 177 patients

Cognitive domain	Test	*n* (%)	Median (IQR)	*n* (%) average	*n* (%) slightly impaired	*n* (%) impaired
Global mental status	MMSE	176 (99.4)	29 (28–30)	147 (83.5)	n.a	29 (16.5)
Executive function	CLOX	174 (98.3)	13 (11–14)	151 (86.8)	n.a	23 (13.2)
Executive function	RWT	172 (97.2)	21 (16–26)	150 (87.2)	11 (6.4)	11 (6.4)
Executive function	TMT-A	168 (94.9)	37.5 (26–54)	133 (79.2)	22 (13.1)	13 (7.7)
Executive function	TMT-B	148 (83.6)	89 (60.5–120)	108 (73.0)	16 (10.8)	24 (16.2)
Executive function	FAB total	173 (97.7)	16 (14–18)	98 (56.6)	30 (17.3)	45 (26.0)
Executive function	WMS-R, digit span backward	172 (97.2)	6 (4–7)	132 (76.7)	34 (19.8)	6 (3.5)
Attention	WMS-R, digit span forward	173 (97.7)	7 (6–8)	137 (79.2)	28 (16.2)	8 (4.6)
	VLMT learning, total score	170 (96.0)	41 (34–51)	129 (75.9)	39 (22.9)	2 (1.2)
	VLMT early recall	171 (96.6)	9 (5–11)	117 (68.4)	47 (27.5)	7 (4.1)
Memory function	VLMT delayed recall	170 (96.0)	8 (6–11)	107 (62.9)	57 (33.5)	6 (3.5)
Memory function	ROCF delayed recall	162 (91.5)	16 (10.5–20.5)	114 (70.4)	16 (9.9)	32 (19.8)
	VLMT recognition	169 (95.5)	12 (8–14)	131 (77.5)	23 (13.6)	15 (8.9)
Visuoconstruction	ROCF copy	168 (94.9)	33 (30–35)	111 (66.1)	17 (10.1)	40 (23.8)
	ROCF immediate recall	163 (92.1)	17 (11.5–21)	121 (74.2)	21 (12.9)	21 (12.9)
	ROCF recognition	159 (89.8)	20 (19–22)	136 (85.5)	10 (6.3)	13 (8.2)

CLOX, clock drawing task, FAB, Frontal Assessment Battery, IQR, interquartile range, MMSE, Mini-Mental State Examination, n.a., not applicable, ROCF, Rey–Osterrieth complex figure test, RWT, Regensburger Wortflüssigkeitstest, TMT A&B, Trail Making Test A&B, VLMT, Verbaler Lern- und Merkfähigkeitstest, WMS-R, Wechsler Memory Scale-Revised

### Factors Associated with Cognitive Outcomes 1 Year After SAH

Patient and disease related factors associated with single cognitive deficits in univariate analysis are given in Supplemental Table 2. In multivariable analysis, delayed cerebral ischemia (adjusted odds ratio [aOR] 3.80, 95% confidence interval [CI] 1.13–12.77, *p* = 0.021), a higher mRS score at discharge (aOR 1.43, 95% CI 1.15–1.77, *p* = 0.001), and fewer years of education (aOR 0.86, 95% CI 0.75–0.98, *p* = 0.019) were associated with more frequent memory deficits 1 year after SAH. Similarly, a higher mRS score at discharge (aOR 1.65, 95% CI 1.33–2.04, *p* < 0.001) and fewer years of education (aOR 0.81, 95% CI 0.70–0.94, *p* = 0.006) predicted the occurrence of executive dysfunctions. Patients with an additional intraparenchymal hemorrhage on the admission CT scan (aOR 3.50, 95% CI 1.35–9.04, *p* = 0.010), loss of consciousness at ictus (aOR 2.57, 95% CI 1.15–5.73, *p* = 0.022), and fewer years of education (aOR 0.75, 95% CI 0.61–0.92, *p* = 0.005) were at higher risk of attention deficits at the 1-year follow-up.

### Cognitive Outcomes in Patients with Perimesencephalic SAH

Of 177 patients, 31 (18%) presented with perimesencephalic SAH. Although not significant, these patients had lower prevalence of cognitive deficits (61%) in comparison with patients with non-perimesencephalic SAH (71%, *p* = 0.277): 42% (vs. 51%, *p* = 0.324) exhibited memory deficits, 23% (vs. 36%, *p* = 0.102) exhibited executive dysfunctions, 23% (vs. 34%, *p* = 0.207) exhibited deficits in visuoconstruction, and 13% (vs. 21%, *p* = 0.329) exhibited deficits in attention.

### Mental Health Outcomes 1 Year After SAH

Depressive and anxiety symptoms (HADS score > 7) were reported by 16% (27 of 165) and 33% (54 of 165) of patients, respectively. In comparison, at SAH onset, 21 (12%) patients were treated for depression and 8 (5%) patients were treated for anxiety. There was no association between premedical history of depression or anxiety and self-reported depressive (*p* = 0.103) or anxiety symptoms (*p* = 1.000) at the 1-year follow-up. Patients with depressive symptoms at the 1-year follow-up more commonly had deficits in memory than nondepressive patients (74% vs. 44%; *p* = 0.006), which was especially true for visual memory (*p* = 0.046) but not for verbal memory (*p* = 0.077). No difference was found for other cognitive domains (all *p* > 0.05). Patients with or without anxiety symptoms had similar frequencies of cognitive deficits (all *p* > 0.05). Premedical history of depression or anxiety had no effect on cognitive deficits (all *p* > 0.05).

### Relationship Between Cognitive/Mental Health Status and Functional Outcome 1 Year After SAH

**Fig. 3:**
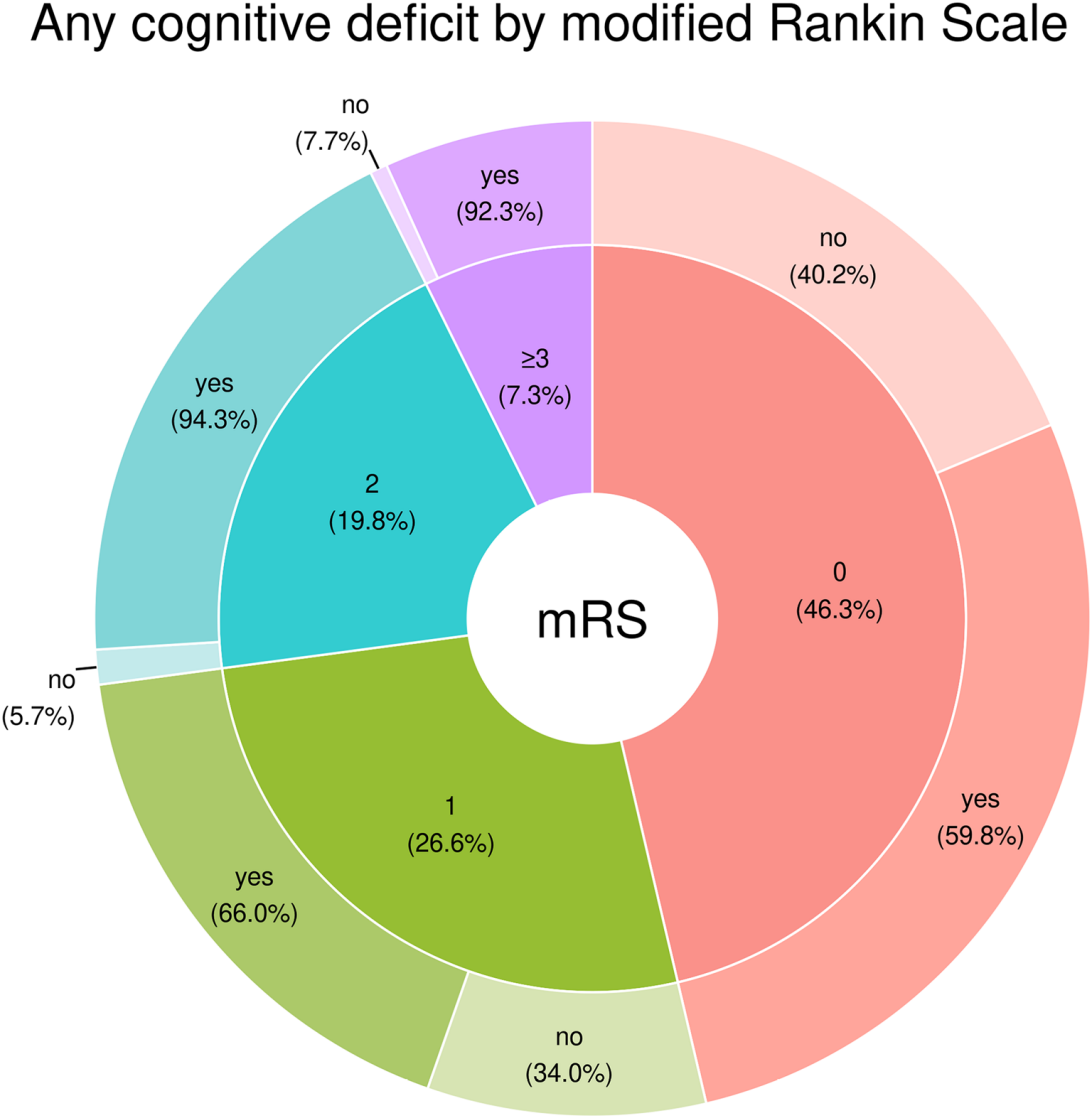
Percentages of cognitive deficits (any domain) across scores on the modified Rankin Scale (mRS) in 177 patients with subarachnoid hemorrhage

Functional outcomes as assessed with the mRS improved from 2 (IQR 1–4) at discharge to 1 (IQR 0–2) 1 year after SAH (*p* < 0.001; Supplemental Fig. 2). Accordingly, 93% achieved good functional 1-year outcome (mRS score 0–2). However, 69% of patients with good functional outcome had a cognitive deficit in at least one cognitive domain (Fig. 3, Supplemental Fig. 3). More specifically, in patients presenting without any functional deficit (mRS score = 0, *n* = 82, 46%), detailed neuropsychological testing revealed cognitive deficits in 60% (*n* = 49), with the following distribution: impairments in memory (37%), visuoconstruction (30%), executive functions (21%), or attention (16%).

Functional outcome 1 year after SAH was affected by deficits in memory (*p* = 0.001) and executive functions (*p* < 0.001) after correcting for the H&H score and age. Impairments in visuoconstruction (*p* = 0.177) and attention (*p* = 0.065) did not influence functional outcome (Table 3). Self-reported symptoms of depression (*p* < 0.001) and anxiety (*p* = 0.028) co-occurred with worse functional outcome irrespective of the H&H score and age (Table 3).

**Table 3:** Associations between cognitive/mental health status and functional outcome 1 year after SAH

Variable	Adjusted OR	95% CI	*p* value	*n*
Any cognitive deficit	2.86	1.47–5.57	0.002	176
Executive deficits	3.23	1.72–6.07	< 0.001	175
Visuoconstructive deficits	1.52	0.83–2.82	0.177	168
Memory deficits	2.71	1.49–4.95	0.001	170
Attention deficits	1.98	0.96–4.09	0.065	172
HADS-A > 7	2.01	1.08–3.74	0.028	165
HADS-D > 7	4.64	2.14–10.10	< 0.001	165

Multivariable regression models were each calculated with generalized linear models with the modified Rankin Scale score at 12 months serving as an ordinal dependent variable. All models were adjusted for the Hunt and Hess score on admission and age as established outcome parameters. One patient with a modified Rankin Scale score of 5 was excluded from analysis.

CI, confidence interval, HADS-A, Hospital Anxiety and Depression Scale anxiety subscale, HADS-D, Hospital Anxiety and Depression Scale depression subscale, OR, odds ratio, SAH, subarachnoid hemorrhage

### HR-QoL 1 Year After SAH

HR-QoL was impaired in 37% (55 of 147) of patients, with 21% reporting restrictions in the physical component summary, 27% reporting restrictions in the mental component summary, and 11% reporting restrictions in both components. Patients with cognitive deficits at the 1-year follow-up (*p* = 0.001) or mental health issues (*p* < 0.001) more frequently reported impaired HR-QoL. Moreover, functional outcome was worse in patients with impaired HR-QoL (*p* < 0.001; Supplemental Table 3).

### Return-to-Work

Based on available data (*n* = 119 of 177), 45% were retired. Of the remaining, 34% returned to their previous occupation, 4% returned but worked fewer hours, 14% failed to return, and 3% were not employed before the bleeding.

## Discussion

We describe multidimensional 1-year outcomes, including detailed domain-specific cognitive outcomes, mental health outcomes, functional outcomes, and QoL measures, from a large cohort of patients with spontaneous SAH who were treated over a time span of ten years. We found that seven of ten patients had evident cognitive deficits, which was highest for memory deficits, followed by deficits in executive functions, visuoconstruction, and attention. Even in patients with excellent functional recovery, cognitive deficits were evident in 61% of cases. The most consistent prognostic factors for impairments across all cognitive domains were worse functional status at ICU discharge and fewer years of education. Every third or sixth patient reported anxiety or depressive symptoms, respectively. Cognitive deficits and restrictions in mental health co-occurred with worse functional outcome and HR-QoL.

The overall cognitive performance was worse than that in the normal population, with a high prevalence of cognitive impairments (71%) in our patients. In comparison to the existing literature, in which prevalence rates range from 40 to 70% after aneurysmal SAH, our results are at the upper end of this range [3]. Interestingly, all tested domains of cognitive function were affected with high rates of co-occurrence, which may reflect a more global injury after SAH. As confirmed by others, the highest rate of domain-specific deficits was related to memory dysfunction in our population (51%), which ranges from 14 to 61% in the literature [5, 6]. The estimated prevalence of executive and attention dysfunctions after SAH varies significantly in previous reports, ranging from 3 to 76%, thus making a comparison challenging [3, 5]. There is a limited body of literature reporting cognitive outcomes after perimesencephalic SAH [23, 24], and only low patient numbers were included in respective studies [25, 26]. One study including 18 patients with perimesencephalic SAH found impairments in at least one cognitive domain in 72% (13 of 18 patients); visual memory was impaired in 39%, immediate memory was impaired in 33%, abstraction was impaired in 33%, and verbal fluency was impaired in 28% [25] between 3 months and 6 years after SAH. Another study reported deficits in attention in 25% (3 of 12 patients), poor or impaired memory in 83% (10 of 12 patients), and executive dysfunctions in 33% 12 months after SAH [26]. Thus, these studies suggest slightly higher prevalence rates of cognitive impairments in comparison with our cohort (any cognitive deficit: 61%; memory deficits: 42%; executive dysfunctions: 23%, deficits in visuoconstruction: 23%; deficits in attention: 13%). However, low patient numbers, as well as differing applied tests and definitions of abnormalities, make comparisons challenging. The high variability of cognitive outcome measures among studies has several reasons, as nonstandardized neuropsychological testing batteries with a heterogeneity of scale-specific metrics and inconsistent cutoff scores, variable timing of the testing, and heterogenous study populations were reported [3, 5]. On the other hand, detailed neuropsychological testing is not feasible in patients with SAH with poor recovery [6]. Our data reflect this selection bias of patients with good functional recovery 1 year after the bleeding. This means that we may have likely underestimated the true prevalence of cognitive impairments after SAH. Still, especially in physically independent patients, it is important to screen for and be alert of cognitive deficits that may interact with day-to-day life and the capacity to return to work because these patients may best benefit from tailored rehabilitative measures.

We found that a worse functional outcome at ICU discharge and fewer years of education were consistent risk factors for all tested domains of cognition. However, as only test scores of the TMT-B were adjusted for education years using published normative values, we cannot exclude the preexistence of cognitive deficits in patients with fewer education years given the fact that lower education years have previously been linked to lower memory performance irrespective of brain injury. Still, our finding is in agreement with previous reports [27, 28] and underlines the advantage of individual patients with an increased cognitive reserve before SAH. A higher mRS score at discharge reflects the sum of early and secondary brain injury together with non-disease-specific adverse events during the ICU course, all of which would in principle be modifiable to some part. Mechanisms of early and secondary brain injury, such as increased intracranial pressure, ischemia, blood–brain barrier breakdown, excitotoxicity, cortical spreading depolarizations, toxic effect of blood degradation products [29, 30], neuroinflammation [31], autoinflammation [32], and mitochondrial dysfunction [33, 34], can trigger diffuse neurodegeneration and axonal injury [30, 35], resulting in focal and diffuse global damage to the brain tissue [5, 36]. It is well accepted that disease severity parameters are associated with cognitive impairments in the long term after SAH [3, 5]. Interestingly, we could not replicate previous results by linking a higher clinical initial disease severity grade with poor cognitive performance. Although significant in univariate analysis, the H&H score did not remain significant in multivariable analysis. This may be explainable be the relatively high proportion of good-grade patients included in our cohort (H&H score 1–3, 81%).

Our findings confirm the debilitating nature of SAH-related cognitive dysfunctions, with negative effects on QoL even in patients with good functional outcome. Cognitive deficits impact patients’ behaviors, such as activities of daily living, social and leisure activities, and the ability to return to work. This qualifies cognitive impairment after SAH as a potential candidate to target in future clinical trials. Promising neuroprotective treatment strategies targeting white matter injury are not yet ready for clinical practice [37]. Still, individualized rehabilitative measures, such as neurocognitive training and reintegration programs, are of importance, which is only feasible if comprehensive neuropsychological examinations are incorporated and patients are made aware of the cognitive consequences after SAH [4]. Patients benefit from tailored neurocognitive rehabilitation programs to improve cognitive function and to learn coping strategies or use compensatory tools [11, 38]. In this regard, it is important to particularly pay attention to mental health symptoms, which are associated with the level of patients’ participation in rehabilitation programs. In accordance with previous literature, we found higher prevalence rates of mental health issues, including depression and anxiety, after SAH than in the general population [5, 39]. Both depression and anxiety were linked to poor QoL and functional outcome in our cohort [5]. Consistent with others, only depression was linked to memory deficits [40].

Some limitations of this study deserve mention. First, we did not include a control group to assess for real-life differences with the general population. However, to overcome this issue, we used age-based normative data for all cognitive test domains. Second, we do not have baseline data before the disease, so we cannot assume that all cognitive impairments in our patients are solely disease related. Still, normative data and the relatively young patient age help to minimize this bias. Third, we do not have longitudinal data to conclude on temporal evolutions of neuropsychological outcomes in our patients. Repeated detailed and time-consuming neuropsychological testing, which would imply the bias of training effects, would not be feasible. We think that the timing of 1 year post injury is useful because most patients have finished intensive rehabilitation and are after their active phase of recovery. A future healthy control group–matched multicenter study should be undertaken to confirm our results. Last, we included patients with aneurysmal and non-aneurysmal SAH, limiting the comparability to other studies in which only patients with aneurysmal SAH were included. We therefore provide a subanalysis for patients with perimesencephalic SAH, who are known to have fewer complications and better long-term outcomes. From a pathophysiological point of view, patients with an aneurysmal pattern of blood distribution on imaging modalities, even without the detection of an aneurysm, have similar complications and therefore similar outcomes as patients with aneurysmal SAH.

## Conclusions

Our study suggests that domain-specific cognitive deficits, specifically memory deficits, together with mental health issues, including anxiety and depression, are common after SAH despite good functional recovery. Both cognitive and mental health deficits had negative effects on HR-QoL measures. This calls for a thorough screening of neurocognitive as well as mental health outcomes in patients with SAH to offer a more tailored rehabilitative program with the aim to improve patients’ QoL and the return-to-work rate. Furthermore, cognitive deficits should serve as an outcome parameter for the ultimate goal to develop neuroprotective treatment.

### Supplementary Information

Below is the link to the electronic supplementary material.

Supplementary file 1 (DOCX 473 KB)

## Data Availability

None.

## Data Availability

The data that support the findings of this study are available from the corresponding author upon reasonable request.
