# Supplementary material for: Cognitive, Mental Health, Functional, and Quality of Life Outcomes 1 Year After Spontaneous Subarachnoid Hemorrhage: A Prospective Observational Study
Source: Neurocrit Care. 2023 Dec 21;41(1):70–9. doi: 10.1007/s12028-023-01895-y (PMC11335887; doi:10.1007/s12028-023-01895-y)
Supplement: Supplementary file 1 — (DOCX 473 KB) [file 12028_2023_1895_MOESM1_ESM.docx]

**Supplemental Figure S1:** Flow chart showing the patient selection.

**
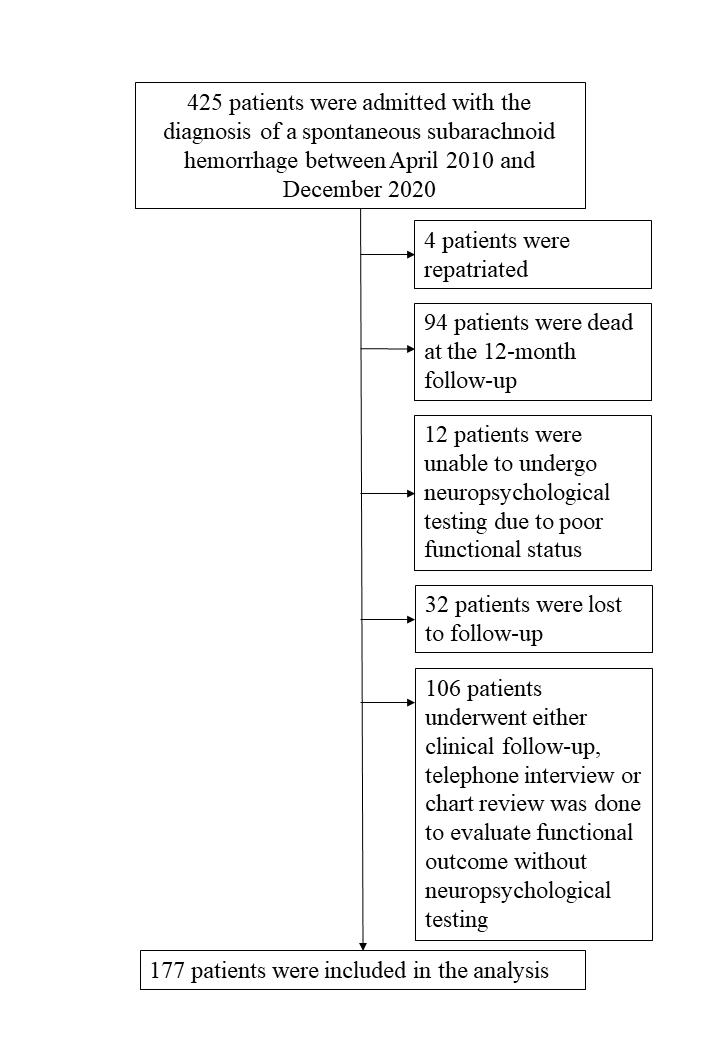
**

**Supplemental Figure S2:** Evolution of the modified Rankin Scale Score (mRS) over one year after subarachnoid hemorrhage.

**
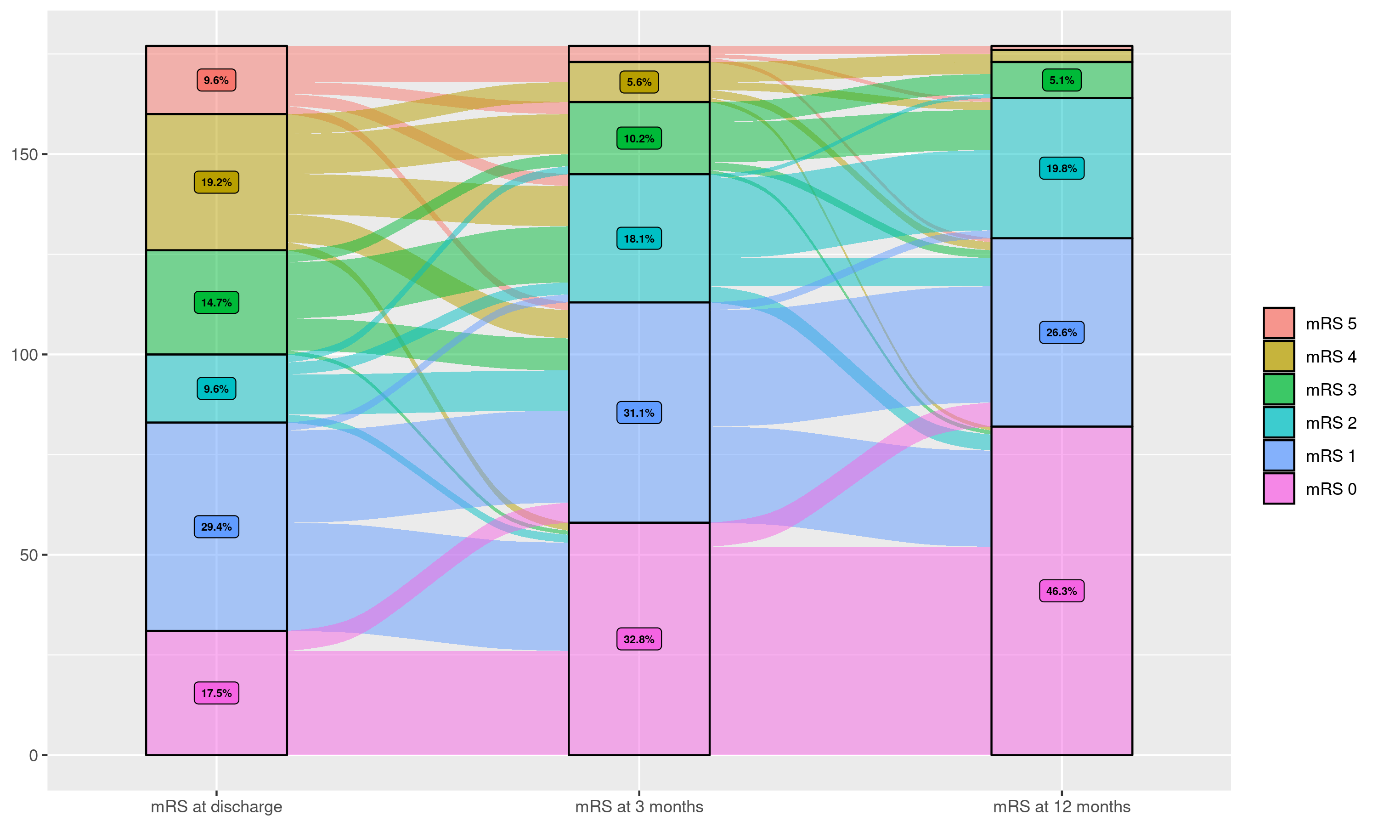
**

**Supplemental Figure S3:** Percentages of deficits in (A) memory, (B) visuoconstruction, (C) executive functions, and (D) attention across scores on the modified Rankin Scale (mRS) in 177 SAH patients.


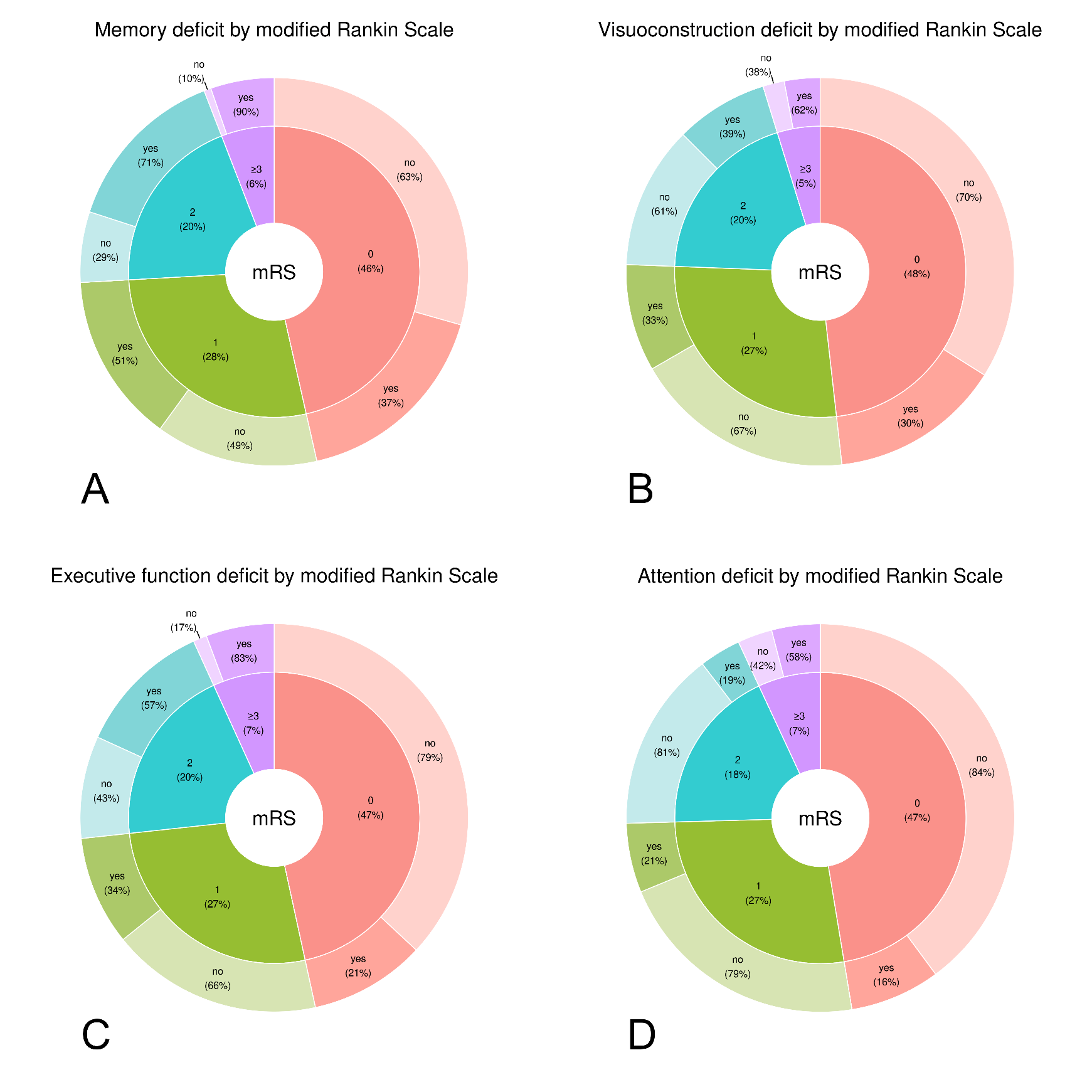
**Supplemental Table S1:** Demographics of included and excluded patients.

|  | Included patients | Excluded patients | p-value |
| --- | --- | --- | --- |
|  | N=177 | N=248 |  |
| **Baseline characteristics** |  |  |  |
| Age [years] | 54 (47 – 62) | 59 (50 – 71) | **<0.001** |
| Female sex | 105 (59.3) | 154 (62.1) | 0.614 |
| Hypertension history | 69 (39) | 92 (37.1) | 0.761 |
| Diabetes mellitus II | 8 (4.5) | 18 (7.3) | 0.306 |
| Smoking history | 72 (40.7) | 61 (25.4) | **<0.001** |
| **Admission variables** |  |  |  |
| Loss of consciousness at ictus | 51 (28.8) | 111 (44.8) | **<0.001** |
| Parenchymal bleeding at admission | 28 (16.1) | 64 (25.8) | **0.023** |
| Hunt and Hess Score at admission | 2 (1 – 3) | 3 (1 – 5) | **<0.001** |
| Modified Fisher Scale at admission | 3 (2 – 4) | 4 (3 – 4) | **0.002** |
| SEBES score at admission | 1 (0 – 2) | 1 (0 – 3) | 0.293 |
| Hjidra score | 11 (6 – 18) | 16 (8 – 23) | **<0.001** |
| Hjidra ventricle score | 1 (0 – 4) | 4 (1 – 6) | **<0.001** |
| **Aneurysm location** |  |  |  |
| Anterior circulation | 83 (46.9) | 126 (50.8) | 0.433 |
| Posterior circulation | 42 (23.7) | 59 (23.8) | 1.000 |
| No aneurysm | 51 (28.8) | 58 (23.4) | 0.217 |
| Unknown/other | 1 (0.6) | 5 (2) | 0.207 |
| **Aneurysm treatment** |  |  |  |
| Coiling | 88 (49.7) | 110 (44.4) | 0.28 |
| Clipping | 39 (22) | 49 (19.8) | 0.326 |
| No intervention | 51 (28.8) | 88 (35.5) | 0.173 |
| **Hospital complications** |  |  |  |
| Hydrocephalus requiring EVD | 71 (40.1) | 129 (52) | **0.018** |
| Large-vessel vasospasm | 81 (45.8) | 102 (41.1) | 0.372 |
| Delayed cerebral ischemia | 22 (12.4) | 42(16.9) | 0.218 |
| Ventriculitis | 21 (11.9) | 19 (7.7) | 0.177 |
| Pneumonia | 61 (34.5) | 94 (37.9) | 0.476 |
| Urinary tract infection | 41 (23.2) | 58 (23.4) | 1.000 |
| Sepsis/Bacteremia | 18 (10.2) | 37 (14.9) | 0.187 |
| **Outcomes** |  |  |  |
| Length of ICU stay [days] | 17 (10 – 27) | 14 (6 – 27) | **0.034** |
| Hospital mortality | 0 (0) | 74 (29.8) | **<0.001** |
| mRS at discharge | 2 (1 – 4) | 5 (2 – 6) | **<0.001** |
| SEBES - subarachnoid hemorrhage early brain edema score; EVD – external ventricular drain; mRS – modified Rankin Scale Score  Data are given in n (%) or median (IQR). Univariate analysis was done with the Fisher’s exact test, T-test or Mann-Whitney U test, as appropriate. | | | |

**Supplemental Table S2:** Univariate associations between patient and disease related factors and one-year cognitive deficits in 177 SAH patients.

| **Variable** | **Memory impaired (n=86/170; 51%)** | **Memory normal (n=84/170; 49%)** | **p-value** |
| --- | --- | --- | --- |
| Age [years] | 55 (47 – 63) | 53 (46 – 60) | 0.101 |
| Years of education | 9 (9 – 11) | 11 (9 – 12) | 0.011 |
| Hypertension history | 39 (45.3) | 27 (32.1) | 0.085 |
| Smoking history | 35 (40.7) | 34 (40.5) | 1.000 |
| Female sex | 51 (59.3) | 50 (59.5) | 1.000 |
| Loss of consciousness at ictus | 23 (26.7) | 26 (31.0) | 0.613 |
| Hunt and Hess Score at admission | 2 (1 – 3) | 2 (1 – 3) | 0.018 |
| Aneurysm location: Anterior circulation | 38 (44.7) | 42 (50.0) | 0.539 |
| Aneurysm location: Posterior circulation | 26 (30.6) | 14 (16.7) | 0.047 |
| No aneurysm | 21 (24.7) | 28 (33.3) | 0.237 |
| Modified Fisher on admission | 3 (2 – 4) | 3 (2 – 4) | 0.726 |
| Parenchymal bleeding at admission | 17 (19.8) | 10 (11.9) | 0.209 |
| SEBES score at admission | 2 (0 – 3) | 1 (0 – 2) | 0.045 |
| Hijdra score | 12.5 (8 – 18) | 10 (4 – 16) | 0.022 |
| Hijdra ventricle score | 2 (0 – 4) | 1 (0 – 4) | 0.069 |
| Coil vs. clip (n=121) | 46 (70.8) vs. 19 (29.2) | 38 (67.9) vs. 18 (32.1) | 0.843 |
| Mechanical ventilation during ICU stay | 63 (73.3) | 48 (57.1) | 0.036 |
| Hydrocephalus req. external ventricular drain | 44 (51.2) | 23 (27.4) | 0.002 |
| Ventriculoperitoneal shunt | 11 (12.8) | 7 (8.3) | 0.456 |
| Large-vessel vasospasm | 41 (47.7) | 38 (45.2) | 0.761 |
| Delayed cerebral ischemia | 18 (20.9) | 4 (4.8) | 0.002 |
| Pneumonia | 38 (44.2) | 20 (23.8) | 0.006 |
| Ventriculitis | 14 (16.3) | 6 (7.1) | 0.094 |
| Sepsis/Bacteremia | 14 (16.3) | 4 (4.8) | 0.023 |
| Length of ICU days | 21 (12 – 31) | 15 (10 – 22) | 0.006 |
| mRS at discharge | 3 (1 – 4) | 1 (0 – 3) | <0.001 |
|  | **Attention impaired (n=36/173; 21%)** | **Attention normal (n=137/173; 79%)** | **p-value** |
| Age [years] | 50 (42 – 59) | 54 (48 – 62) | 0.109 |
| Years of education | 9 (8 – 11) | 11 (9 – 12) | 0.003 |
| Hypertension history | 13 (36.1) | 56 (40.9) | 0.703 |
| Smoking history | 15 (41.7) | 55 (40.1) | 1.000 |
| Female sex | 21 (58.3) | 81 (59.1) | 1.000 |
| Loss of consciousness at ictus | 17 (47.2) | 33 (24.1) | 0.012 |
| Hunt and Hess Score at admission | 2 (2 – 4) | 2 (1 – 3) | 0.048 |
| Aneurysm location: Anterior circulation | 17 (47.2) | 63 (46.3) | 1.000 |
| Aneurysm location: Posterior circulation | 9 (25.0) | 33 (24.3) | 1.000 |
| No aneurysm | 10 (27.8) | 40 (29.4) | 0.360 |
| Modified Fisher on admission | 3 (2 – 4) | 3 (2 – 4) | 0.405 |
| Parenchymal bleeding at admission | 11 (30.6) | 17 (12.4) | 0.019 |
| SEBES score at admission | 2 (0 – 3) | 1 (0 – 2) | 0.248 |
| Hijdra score | 12 (9.25 – 16.75) | 11 (5.25 – 17.75) | 0.420 |
| Hijdra ventricle score | 1.5 (0 – 4) | 1 (0 – 4) | 0.709 |
| Coil vs. clip (n=123) | 18 (69.2) vs. 8 (30.8) | 67 (69.1) vs. 30 (30.9) | 1.000 |
| Mechanical ventilation during ICU stay | 25 (69.4) | 88 (64.2) | 0.694 |
| Hydrocephalus req. external ventricular drain | 16 (44.4) | 53 (38.7) | 0.569 |
| Ventriculoperitoneal shunt | 3 (8.3) | 15 (10.9) | 0.768 |
| Large-vessel vasospasm | 17 (47.2) | 62 (45.3) | 0.853 |
| Delayed cerebral ischemia | 8 (22.2) | 13 (9.5) | 0.047 |
| Pneumonia | 12 (33.3) | 46 (33.6) | 1.000 |
| Ventriculitis | 6 (16.7) | 15 (10.9) | 0.390 |
| Sepsis/Bacteremia | 4 (11.1) | 13 (9.5) | 0.757 |
| Length of ICU days | 18 (10 – 30) | 17 (11 – 26) | 0.855 |
| mRS at discharge | 2 (1 – 4) | 2 (1 – 4) | 0.624 |
|  | **Executive function impaired (n=63/176; 36%)** | **Executive function normal (n=113/176; 64%)** | **p-value** |
| Age [years] | 54 (48 – 63) | 54 (46 – 60) | 0.427 |
| Years of education | 9 (8 – 11) | 11 (9 – 12) | 0.008 |
| Hypertension history | 30 (47.6) | 39 (34.5) | 0.108 |
| Smoking history | 32 (50.8) | 39 (34.5) | 0.039 |
| Female sex | 41 (65.1) | 64 (56.6) | 0.337 |
| Loss of consciousness at ictus | 26 (41.3) | 25 (22.1) | 0.009 |
| Hunt and Hess Score at admission | 3 (2 – 4) | 2 (1 – 3) | <0.001 |
| Aneurysm location: Anterior circulation | 28 (44.4) | 55 (49.1) | 0.638 |
| Aneurysm location: Posterior circulation | 22 (34.9) | 20 (17.9) | 0.016 |
| No aneurysm | 13 (20.6) | 37 (33.0) | 0.116 |
| Modified Fisher on admission | 3 (2 – 4) | 3 (2 – 4) | 0.165 |
| Parenchymal bleeding at admission | 13 (20.6) | 15 (13.3) | 0.206 |
| SEBES score at admission | 2 (0 – 3) | 1 (0 – 2) | 0.010 |
| Hijdra score | 13 (9 – 19) | 10 (4 – 16) | 0.035 |
| Hijdra ventricle score | 2 (0 – 5) | 1 (0 – 3) | 0.047 |
| Coil vs. clip (n=126) | 32 (64) vs. 18 (36) | 56 (73.7) vs. 20 (26.3) | 0.321 |
| Mechanical ventilation during ICU stay | 48 (76.2) | 68 (60.2) | 0.033 |
| Hydrocephalus req. external ventricular drain | 37 (58.7) | 34 (30.1) | <0.001 |
| Ventriculoperitoneal shunt | 10 (15.9) | 10 (8.8) | 0.215 |
| Large-vessel vasospasm | 37 (58.7) | 44 (38.9) | 0.018 |
| Delayed cerebral ischemia | 9 (14.3) | 13 (11.5) | 0.638 |
| Pneumonia | 30 (47.6) | 30 (26.5) | 0.008 |
| Ventriculitis | 13 (20.6) | 8 (7.1) | 0.014 |
| Sepsis/Bacteremia | 8 (12.7) | 10 (8.8) | 0.444 |
| Length of ICU days | 25 (16 – 33) | 16 (10 – 23) | <0.001 |
| mRS at discharge | 3 (1 – 4) | 1 (1 – 3) | <0.001 |
| SEBES - subarachnoid hemorrhage early brain edema score; mRS – modified Rankin Scale Score  Data are given in n (%) or median (IQR). Univariate analysis was done with the Fisher’s exact test, T-test or Mann-Whitney U test, as appropriate. | | | |

**Supplemental Table S3:** Univariate associations between multidimensional one-year outcomes and one-year health-related quality of life in 147 SAH patients.

| **Supplemental Table 4: Univariate associations between multidimensional one-year outcomes and one-year health-related quality of life in 147 SAH patients.** | | | | | | | | | |
| --- | --- | --- | --- | --- | --- | --- | --- | --- | --- |
|  | **MCS <40** | **MCS ≥40** | **p-value** | **PCS <40** | **PCS ≥40** | **p-value** | **MCS or PCS <40** | **MCS or PCS ≥40** | **p-value** |
| **Mental health impairments** | | | | | | | | | |
| **HADS-A >7** | 33 (83) | 16 (15) | **<0.001** | 15 (48) | 34 (30) | 0.058 | 36 (66) | 13 (14) | **<0.001** |
| **HADS-D >7** | 19 (48) | 5 (5) | **<0.001** | 10 (32) | 14 (12) | **0.013** | 21 (38) | 3 (3) | **<0.001** |
| **Cognitive deficits** | | | | | | | | | |
| **Any domain** | 33 (83) | 65 (61) | **0.018** | 27 (87) | 71 (61) | **0.009** | 46 (84) | 52 (57) | **0.001** |
| **Executive deficits** | 15 (38) | 28 (26) | 0.222 | 14 (45) | 29 (25) | **0.044** | 21 (38) | 22 (24) | 0.091 |
| **Visuoconst-ructive deficits** | 13 (33) | 30 (29) | 0.682 | 10  (33) | 33 (29) | 0.658 | 19 (35) | 24 (27) | 0.347 |
| **Memory deficits** | 27 (67) | 41 (39) | **0.003** | 19 (61) | 49 (43) | 0.104 | 35 (64) | 33 (37) | **0.002** |
| **Attention deficits** | 6 (15) | 17 (16) | 1.000 | 6 (20) | 17 (15) | 0.574 | 7 (13) | 16 (17) | 0.639 |
| **Functional outcomes** | | | | | | | | | |
| **mRS** | 1 (1-2) | 0 (0-1) | **<0.001** | 2 (1-2) | 0 (0-1) | **<0.001** | 1 (1-2) | 0 (0-1) | **<0.001** |
| MCS – mental component summary; PCS – physical component summary; HADS – Hospital Anxiety and Depression Scale; mRS – modified Rankin Scale Score  Data are given in n (%) or median (IQR). Univariate analysis was done with the Fisher’s exact test or Mann-Whitney U test, as appropriate. | | | | | | | | | |
